# Blood Flow Energy Identifies Coronary Lesions Culprit of Future Myocardial Infarction

**DOI:** 10.1007/s10439-023-03362-3

**Published:** 2023-09-21

**Authors:** Maurizio Lodi Rizzini, Alessandro Candreva, Valentina Mazzi, Mattia Pagnoni, Claudio Chiastra, Jean-Paul Aben, Stephane Fournier, Stephane Cook, Olivier Muller, Bernard De Bruyne, Takuya Mizukami, Carlos Collet, Diego Gallo, Umberto Morbiducci

**Affiliations:** 1 PolitoBIOMed Lab, Department of Mechanical and Aerospace Engineering, Politecnico di Torino, Corso Duca degli Abruzzi 24, 10129 Turin, Italy; 2 Department of Cardiology, Zurich University Hospital, Zurich, Switzerland; 3 Department of Cardiology, Lausanne University Hospital, Lausanne, Switzerland; 4 Pie Medical Imaging BV, Maastricht, The Netherlands; 5 Department of Advanced Biomedical Sciences, University of Naples Federico II, Naples, Italy; 6 Department of Cardiology, HFR Fribourg, Fribourg, Switzerland; 7 Cardiovascular Center Aalst, OLV-Clinic, Aalst, Belgium

**Keywords:** Computational fluid dynamics, Kinetic energy, Rotational energy, Myocardial infarction, Quantitative coronary angiography, Fractional flow reserve, Wall shear stress

## Abstract

The present study establishes a link between blood flow energy transformations in coronary atherosclerotic lesions and clinical outcomes. The predictive capacity for future myocardial infarction (MI) was compared with that of established quantitative coronary angiography (QCA)-derived predictors. Angiography-based computational fluid dynamics (CFD) simulations were performed on 80 human coronary lesions culprit of MI within 5 years and 108 non-culprit lesions for future MI. Blood flow energy transformations were assessed in the converging flow segment of the lesion as ratios of kinetic and rotational energy values (KER and RER, respectively) at the QCA-identified minimum lumen area and proximal lesion sections. The anatomical and functional lesion severity were evaluated with QCA to derive percentage area stenosis (%AS), vessel fractional flow reserve (vFFR), and translesional vFFR (ΔvFFR). Wall shear stress profiles were investigated in terms of topological shear variation index (TSVI). KER and RER predicted MI at 5 years (AUC = 0.73, 95% CI 0.65–0.80, and AUC = 0.76, 95% CI 0.70–0.83, respectively; *p* < 0.0001 for both). The predictive capacity for future MI of KER and RER was significantly stronger than vFFR (*p* = 0.0391 and *p* = 0.0045, respectively). RER predictive capacity was significantly stronger than %AS and ΔvFFR (*p* = 0.0041 and *p* = 0.0059, respectively). The predictive capacity for future MI of KER and RER did not differ significantly from TSVI. Blood flow kinetic and rotational energy transformations were significant predictors for MI at 5 years (*p* < 0.0001). The findings of this study support the hypothesis of a biomechanical contribution to the process of plaque destabilization/rupture leading to MI.

**Supplementary Information:**

The online version contains supplementary material available at 10.1007/s10439-023-03362-3.

## Introduction

Myocardial infarction (MI) represents the leading cause of cardiovascular morbidity and mortality in Western countries [1]. Progression and rupture of atherosclerotic plaques is the most common mechanism leading to MI [2]. Consequently, the identification of atherosclerotic lesions prone to cause MI has assumed prominent clinical relevance.

While the anatomical assessment of lesion severity visually or per quantitative coronary angiography (QCA), usually expressed in terms of percentage area stenosis (%AS), still holds a central role in the clinical decision making [25], intravascular functional evaluation per fractional flow reserve (FFR) [24] has emerged as an objective and reliable tool [40], resulting in improved clinical outcomes [34]. However, there is evidence to show that untreated non-flow limiting lesions can continue to progress and lead to major adverse cardiac events [30]. This indicates the presence of mechanisms contributing to unfavorable prognosis that may not be fully discernible based on intravascular pressure gradients alone.

According to Bernoulli's principle, in an idealized cylindrical conduit with a lumen narrowing, an increase in the velocity of a fluid and the consequent increase in its kinetic energy is balanced by a decrease in static pressure. In the real-world scenario of coronary atherosclerotic lesions, Bernoulli’s principle only partially describes the measured translesional pressure drop [4]. Moreover, the energy transformation resulting from the narrowing of the coronary lumen, which imparts convective acceleration to blood flow, is expected to increase shearing, and might lead to a production of rotational energy [8].

The blood pressure gradient resulting from lumen narrowing is involved not only in the process of energy conversion/transformation when blood flows through the coronary lesion, but vicariously contributes to determine the biomechanical stimuli acting at the blood–endothelium interface, quantifiable in terms of wall shear stress (WSS).

In the context of coronary circulation, computational fluid dynamics (CFD) is gaining momentum in cardiology [5] as it has been used to identify WSS profiles associated with plaque progression and destabilization [14, 39], as well as to predict MI [6, 15, 33, 36]. Recent studies have highlighted that the WSS distribution represents the fingerprint of the hemodynamic complexity developing within the vessel, which in turn is dictated by anatomy, lesion morphology and can be characterized in terms of blood flow vorticity and helicity [8, 22, 23]. Thus, previous evidence points to a possible synergistic relationship between lesion anatomy (lumen geometry and underlying plaque), translesional pressure profiles, WSS, and blood flow energy. However, although some of these factors have been studied in terms of the predictive capacity for adverse events, a flow energy-based analysis with a direct clinical outcome in coronary lesions is still lacking.

Motivated by these observations, the present study aims to investigate the predictive capacity of coronary blood flow energy transformations to identify lesions at risk of causing MI within 5 years based on CFD simulations obtained from conventional coronary angiography. The overarching hypothesis of the study is that the relative narrowing of the proximal segment of a lesion as well as the energy conversions/transformations imparted to coronary blood flow running through it are effective in identifying lesions prone to cause MI. Our hypothesis also posits that such blood flow energy conversions/transformations establishing within lesions can be distilled into WSS profiles that have been recently identified as strong predictors of lesions culprit of future MI [6].

## Methods

### Study Population

The population of this retrospective longitudinal multicentric study was screened aiming to identify anatomic and functional predictors of future MI. The study included three European centers: OLV clinic, Aalst, Belgium; University Hospital of Lausanne CHUV, Switzerland; and Fribourg Cantonal Hospital, Switzerland. As detailed in a previous study [6], lesions culprit of acute MI were clinically identified from coronary angiography in patients retrospectively selected according to the following inclusion criteria: patients with a previous coronary angiography (performed from 1 month to 5 years before MI) presenting with a mild lesion (≤ 50% diameter stenosis) identified by visual inspection, culprit for the future MI (FC), and (at least) one visually identifiable, non-culprit for the future MI (NFC) lesion in one of the other epicardial coronary arteries. The most recent angiography was selected for analysis, in case of multiple coronary angiographies before the acute event. Main exclusion criteria were the presence of coronary bypass graft (CABG) or stent, bifurcation lesions, and insufficient quality of coronary angiography images [6]. Patients were further stratified in patients presenting ST-elevation MI (STEMI) and non-ST-elevation MI (NSTEMI).

The study protocol conforms to the ethical guidelines of the 1975 Declaration of Helsinki and has been approved by the Institutions' ethics committees. Written informed consent was obtained from each patient included in the study.

Starting from 6885 patients who underwent coronary angiography for acute MI from January 2009 to December 2019, 775 had a previous coronary angiography, and 80 (*n* = 188 vessels, 2.35 ± 0.48 vessel/patient) were included in this study according to inclusion and exclusion criteria. The baseline characteristics of the patients, which have been previously presented elsewhere [6], are summarized in Supplementary Table 1 of the Supplementary Materials.

### Quantitative Coronary Angiography and Lesion Geometry Characterization

**Fig. 1:**
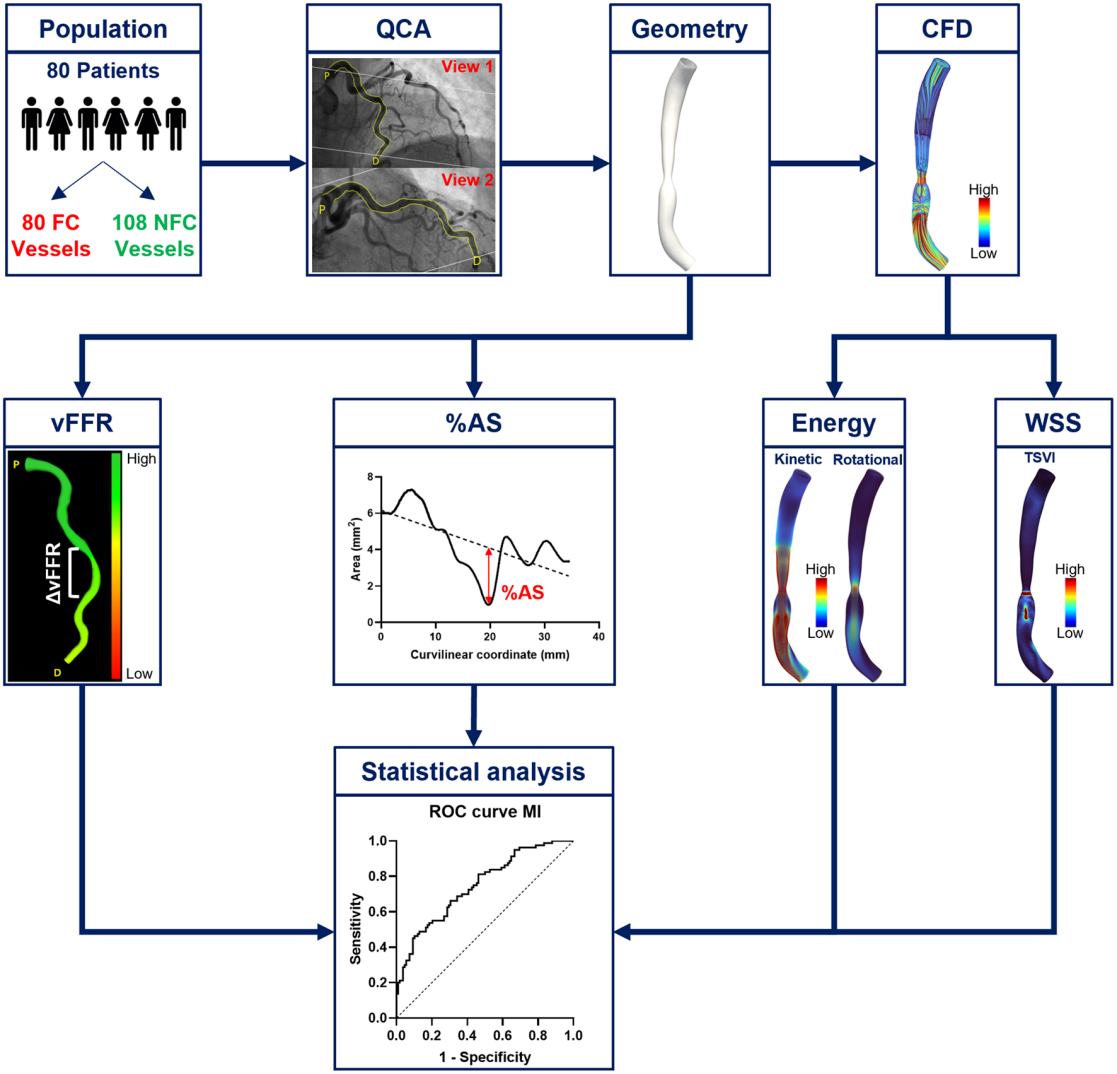
Workflow of the study. From a population of 80 patients, 80 coronary lesions site of myocardial infarction (MI) within 5 years (future culprit) were compared to 108 non-culprit lesions for future MI. The three-dimensional coronary vessel geometries were reconstructed from quantitative coronary angiography (QCA) images and used to obtain anatomical (%AS) and functional (vFFR, ΔvFFR) clinical indicators of coronary disease, and to perform computational fluid dynamics (CFD) simulations. From CFD simulations: (i) wall shear stress was quantified in terms of topological shear variation index (TSVI); (ii) blood flow energy transformations in the converging segment of the lesion were quantified in terms of ratio between kinetic (KER) or rotational energies (RER) at MLA and proximal luminal sections of the lesion. The predictive power for future MI of %AS, vFFR, ΔvFFR, and TSVI was evaluated by means of receiver operating characteristic (ROC) curves and compared with the predictive power of KER and RER

The workflow of the study is reported in Fig. 1. The analysis was performed blinded to the information of which lesion evolved toward MI [6]. The three-dimensional (3D) geometry of each coronary vessel was reconstructed from two coronary angiography projections with at least 30° apart using the CAAS Workstation WSS software (Pie Medical Imaging, Maastricht, the Netherlands) [6]. A detailed description of the 3D vessel reconstruction process is reported in the Supplementary Materials. The reconstruction method has been validated both in vitro [13, 27] and in vivo, by comparing angiography-based reconstructions with those obtained using intravascular ultrasound imaging [28].

**Fig. 2:**
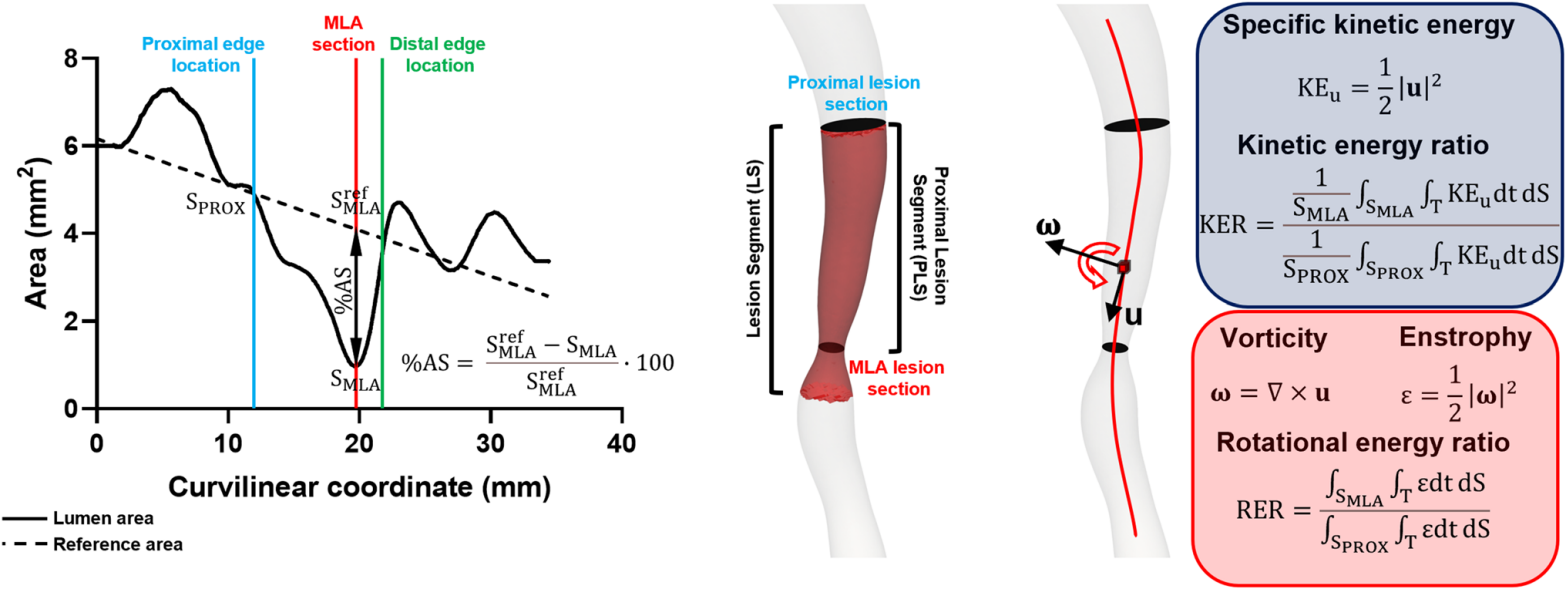
Left panel: QCA-based definition of the atherosclerotic lesion, with proximal and distal lesion cross-sections identified by the intersection between measured lumen area curve and linear regression identifying the reference interpolated lumen area, while minimum lumen area (MLA) section was identified as the section presenting the lowest surface area value inside the lesion segment (LS), defined as the segment between proximal and distal lesion luminal sections. The proximal lesion segment (PLS) was defined as the segment identified by proximal and MLA luminal sections. Right panel: schematic representation of velocity (**u**) and vorticity (**ω**) vectors for a fluid element, and mathematical definition of specific kinetic energy (KE_u_), kinetic energy ratio (KER), **ω**, enstrophy (ε), and rotational energy ratio (RER)

The lesion segment (LS) was unambiguously defined as the segment including the minimum lumen area (MLA) section of the vessel and automatically delimited proximally and distally by the intersection of the 3D-QCA area function line with the interpolated reference line [6] (Fig. 2). According to the clinical practice, the anatomical severity of the lesion was quantified in terms of %AS at the MLA as follows:

1 $$\mathrm{\%AS}= \frac{\mathrm{S}_{\mathrm{MLA}}^{\mathrm{ref}}-\mathrm{S}_{\mathrm{MLA}}}{\mathrm{S}_{\mathrm{MLA}}^{\mathrm{ref}}}\times 100,$$

where $\mathrm{S}_{\mathrm{MLA}}^{\mathrm{ref}}$ is the surface area value of the interpolated reference line at the curvilinear coordinate of the MLA, and $\mathrm{S}_{\mathrm{MLA}}$ is the surface area value of the MLA cross-section of the coronary vessel.

### Computational Hemodynamics

The reconstructed 3D geometries of the coronary vessels were discretized using the commercial software ICEM CFD (Ansys Inc., Canonsburg, PA, USA). Technically, mesh grids with tetrahedral elements in the bulk (with an element edge size ranging from 0.04 to 0.16 mm, and a curvature-based refinement) and five layers of prismatic elements near the wall were generated. The sizing of the computational grid was set based on a mesh grid independence analysis performed in a previous study [23].

The governing equations of fluid motion, the Navier–Stokes equations, were solved in their discretized form under unsteady flow conditions using the finite volume-based CFD code Fluent (Ansys Inc., Canonsburg, PA, USA). Blood was considered as continuous, incompressible (*ρ* = 1060 kg/m^3^), and non-Newtonian fluid. In detail, the shear-thinning rheological behavior of blood was modeled adopting the Carreau fluid model [7]:

2 $$\upmu (\dot{\upgamma )}= {\upmu }_{\infty }+\left({\upmu }_{0}-{\upmu }_{\infty }\right){\left(1+{\left(\uplambda \dot{\upgamma }\right)}^{2}\right)}^{\frac{n-1}{2}}$$

where μ_∞_ is the nominal high shear rate viscosity (i.e., the viscosity value in the shear rate range where blood behaves as a Newtonian fluid), set equal to 0.0035 Pa s, μ_0_ = 0.25 Pa s, λ =  25 s, and n = 0.25 [7]. In Eq. (2), the shear rate $\dot{\upgamma }$ is calculated according to

3 $$\dot{\upgamma }=\sqrt{2\mathrm{D}\left({\bf{u}}\right) :\mathrm{D}({\bf{u}})}=\sqrt{2\mathrm{tr}\left(\mathrm{D}{\left({\bf{u}}\right)}^{2}\right)}$$

where **u** is the velocity vector, and $\mathrm{D}\left({\bf{u}}\right)=\frac{\nabla {\bf{u}}+\nabla {{\bf{u}}}^{\mathrm{T}}}{2}$ is the rate of deformation tensor.

Vessel wall was assumed as rigid. The no-slip condition was applied at wall boundaries. Boundary conditions were applied based on a previous study [6]. In detail, generic (but artery-specific, i.e., typical for left anterior descending, left circumflex, and right coronary artery, Fig. S1 in the Supplementary Material) Doppler velocity recordings [6] were personalized to the specific patient according to a diameter-based scaling law [12] and prescribed in terms of parabolic velocity profile at the inflow section of each vessel [17]. A reference pressure was prescribed at the outflow section. Regarding the applied numerical scheme, second-order accuracy was set for both momentum and pressure equations, which were coupled using a full implicit scheme. The implicit second-order backward Euler scheme was adopted for time integration, with a fixed time step (*N* = 90). The time duration of the cardiac cycle was the same for all the simulated coronary arteries (0.9 s). Convergence was considered achieved when mass and momentum residuals fell below 10^−5^.

### Hemodynamic Characterization

Angiography-derived vessel fractional flow reserve (vFFR) at each point of the vessel was obtained using the commercial software CAAS Workstation vFFR software (Pie Medical Imaging). The 3D QCA-derived vessel FFR has been demonstrated to exhibit a high diagnostic accuracy to detect FFR in the FAST study, where it has been validated in vitro against CFD data and in vivo against pressure wire FFR intravascular measurements [18]. Exhaustive details on the 3D QCA-derived vFFR can be found elsewhere [18]. The translesional vFFR (ΔvFFR) was defined as the difference between vFFR values at the proximal and at the distal edges of the lesion.

Referring to the geometry of a coronary artery with a lesion to the simple fluid dynamics scheme of a conduit with a converging flow segment followed by a diverging one, and inspired by the Bernoulli’s principle, here we analyzed the hemodynamics inside the lesion in terms of blood flow kinetic energy. The specific kinetic energy (KE_u_), i.e., the kinetic energy per mass unit of blood was defined as

4 $${\mathrm{KE}}_{\mathrm{u}}=\frac{1}{2}{\left|{\bf{u}}\right|}^{2},$$

here, the kinetic energy per mass unit of blood produced within the converging flow segment of the lesion along the cardiac cycle was quantified defining the kinetic energy ratio (KER):

5 $$\mathrm{KER}=\frac{\frac{1}{{\mathrm{S}}_{\mathrm{MLA}}}\underset{{\mathrm{S}}_{\mathrm{MLA}}}{\int }\underset{{\mathrm{T}}}{\int }{\mathrm{KE}}_{\mathrm{u}} \; \mathrm{dt}\,\mathrm{dS}}{\frac{1}{{\mathrm{S}}_{\mathrm{PROX}}}\underset{{\mathrm{S}}_{\mathrm{PROX}}}{\int }\underset{{\mathrm{T}}}{\int}{\mathrm{KE}}_{\mathrm{u}} \; \mathrm{dt}\,\mathrm{dS}},$$

where T is the duration of a cardiac cycle, $\mathrm{{S}}_{\mathrm{MLA}}$ is the surface area at the MLA cross-section, and $\mathrm{{S}}_{\mathrm{PROX}}$ is the surface area at proximal cross-section of the lesion (i.e., an ostensibly not diseased section, identified according to Fig. 2).

The energy-based characterization of coronary blood flow was enriched by considering the amount of rotational energy produced because of the presence of the lesion. To do that, the fluid mechanics quantity vorticity, namely the curl of the velocity vector, was considered:

6 $${{\varvec{\upomega}}=\nabla \times {\bf{u}}}$$

Vorticity vector **ω** is a measure of the local spinning motion of the fluid, its local direction indicating the spin axis of rotation (Fig. 2). The strength of the vorticity vector field without directionality implication can be expressed using the scalar quantity enstrophy $\mathsf{\varepsilon}$, defined as

7 $$\mathsf{\varepsilon} =\frac{1}{2}{\left|{\varvec{\upomega}}\right|}^{2}$$

Finally, similarly to the specific kinetic energy, the production of the specific rotational energy caused by the presence of lumen narrowing was quantified introducing the rotational energy ratio (RER):

8 $$\mathrm{RER}=\frac{\underset{{\mathrm{S}}_{\mathrm{MLA}}}{\int }\underset{{\mathrm{T}}}{\int }\mathsf{\varepsilon} \; \mathrm{dt}\, \mathrm{dS}}{\underset{{\mathrm{S}}_{\mathrm{PROX}}}{\int }\underset{{\mathrm{T}}}{\int}\mathsf{\varepsilon} \; \mathrm{dt} \, \mathrm{dS}} $$

The hemodynamic characterization of the lesion was completed considering WSS. In particular, the topological shear variation index (TSVI) averaged over the luminal surface of the lesion was quantified, as it has been recently demonstrated to be a strong predictor of coronary lesions culprit of future MI in the population subject of this study [6]. The WSS divergence-based quantity TSVI [19, 21], identifying peculiar features in the WSS topological skeleton dynamics [19, 21], measures the variability of the expansion/contraction action exerted by the WSS on the endothelium during the cardiac cycle. TSVI is defined as:

9 $$\mathrm{TSVI}= {\left\{\frac{1}{\mathrm{T}}\int_{\mathrm{T}}{\left[\nabla \cdot \left(\frac{{\varvec{\tau}}}{\left|{\varvec{\tau}}\right|}\right)-{\overline{\nabla \cdot \left(\frac{{\varvec{\tau}}}{\left|{\varvec{\tau}}\right|}\right)}}\right]}^{2}\mathrm{dt}\right\}}^{1/2},$$

where ${\varvec{\tau}}$ is WSS vector field and the overbar denotes a cycle-average quantity.

### Statistical Analysis

Continuous variables are presented as median [interquartile range (IQR)]. Mann-Whitney’s *U* test was used to compare differences of continuous variables between FC and NFC lesions. The predictive power of each variable was assessed by receiver operating characteristic (ROC) curves in terms of area under the curve (AUC) and DeLong test [9]. Youden's J statistic was applied to infer the best cut-off from the ROC curves [41]. Accordingly, Kaplan–Meier curves were built from the obtained cut-off values to perform a time-to-event analysis. The strength of the association of blood flow energy-based quantities with %AS, vFFR, ΔvFFR, and TSVI was evaluated in terms of Spearman’s rank correlation coefficient (r). All statistical analyses were performed using R 4.2.1 statistical software (R Foundation for Statistical Computing, Vienna, Austria) assuming a statistical significance for *p* < 0.05.

## Results

### Coronary Blood Flow Energy and Geometric Characterization

**Fig. 3:**
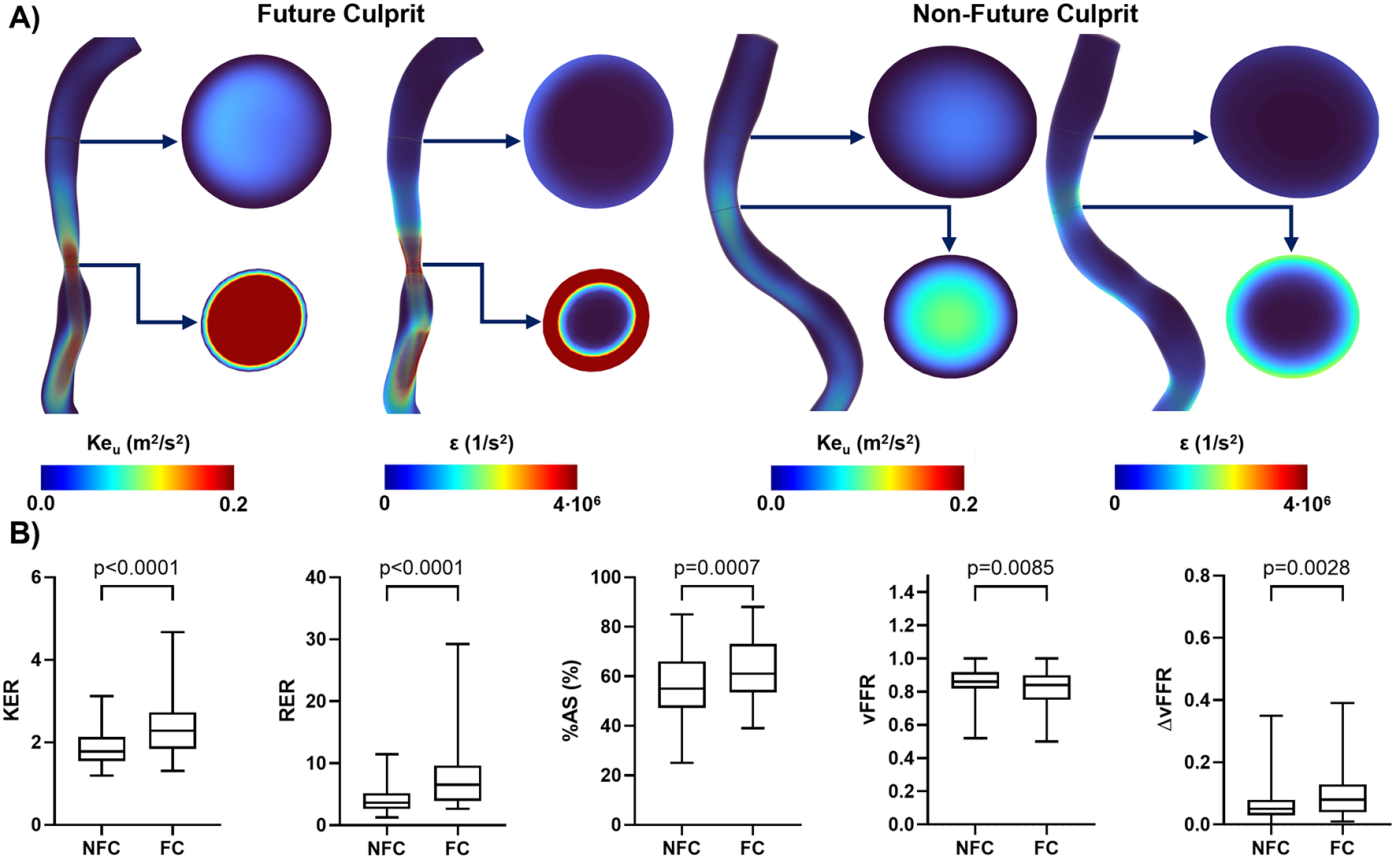
**A** Volume visualizations of cycle-average specific kinetic energy (KE_u_) and enstrophy (ε) in two explanatory lesion types, one future culprit and one non-future culprit. The distributions of cycle-average KE_u_ and ε on the proximal and minimum lumen area sections are also presented. **B** Box plots of KER, RER, %AS, vFFR, and ΔvFFR comparing future culprit (FC) and non-future culprit (NFC) lesions (statistically significant differences between FC and NFC lesions are reported in terms of *p*-values). The *p*-values are reported for each quantity

The distributions of KE_u_ and ε values averaged over the cardiac cycle were visually inspected within all the vessels as well as on proximal and MLA sections. What emerged is presented in Fig. 3A for two representative cases, one FC and one NFC lesions from the same patient. In general, FC lesions hemodynamics was characterized by higher cycle-average kinetic energy and enstrophy values than NFC lesions. Comparing KE_u_ and ε cycle-average values on lesion sections, it was observed that FC and NFC lesions exhibited very similar values on the proximal section but not on the MLA section, where FC lesions were characterized by higher kinetic energy and enstrophy than NFC lesions (Fig. 3A). In terms of cross-sectional distribution of the cycle-average quantities, cycle-average KE_u_ values were always high in the bulk of the vessel (where blood velocities are expected to be higher than near-wall). On the opposite, cycle-average ε values were higher in the near-wall region, where blood velocity gradients are expected to be high, independent of the lesion classification.

Results of the distribution analysis of blood flow energy are presented in Fig. 3B. FC lesions exhibited kinetic and rotational energy profiles significantly different from NFC. In detail, KER (2.28 [1.84–2.73] *vs.* 1.78 [1.55–2.13], *p* < 0.0001) and RER (6.59 [3.92–9.63] *vs.* 3.67 [2.60–5.16], *p* < 0.0001) were significantly higher in FC than NFC lesions. A significant difference between FC and NFC lesions emerged also for %AS (61.00 [53.50–73.00] *vs.* 55.00 [47.25–66.00], *p* = 0.0007), vFFR (0.84 [0.75–0.90] *vs.* 0.86 [0.82–0.92], *p* = 0.0085), and ΔvFFR (0.08 [0.04–0.13] vs. 0.05 [0.03–0.08], *p* = 0.0028).

### Myocardial Infarction Prediction

**Fig. 4:**
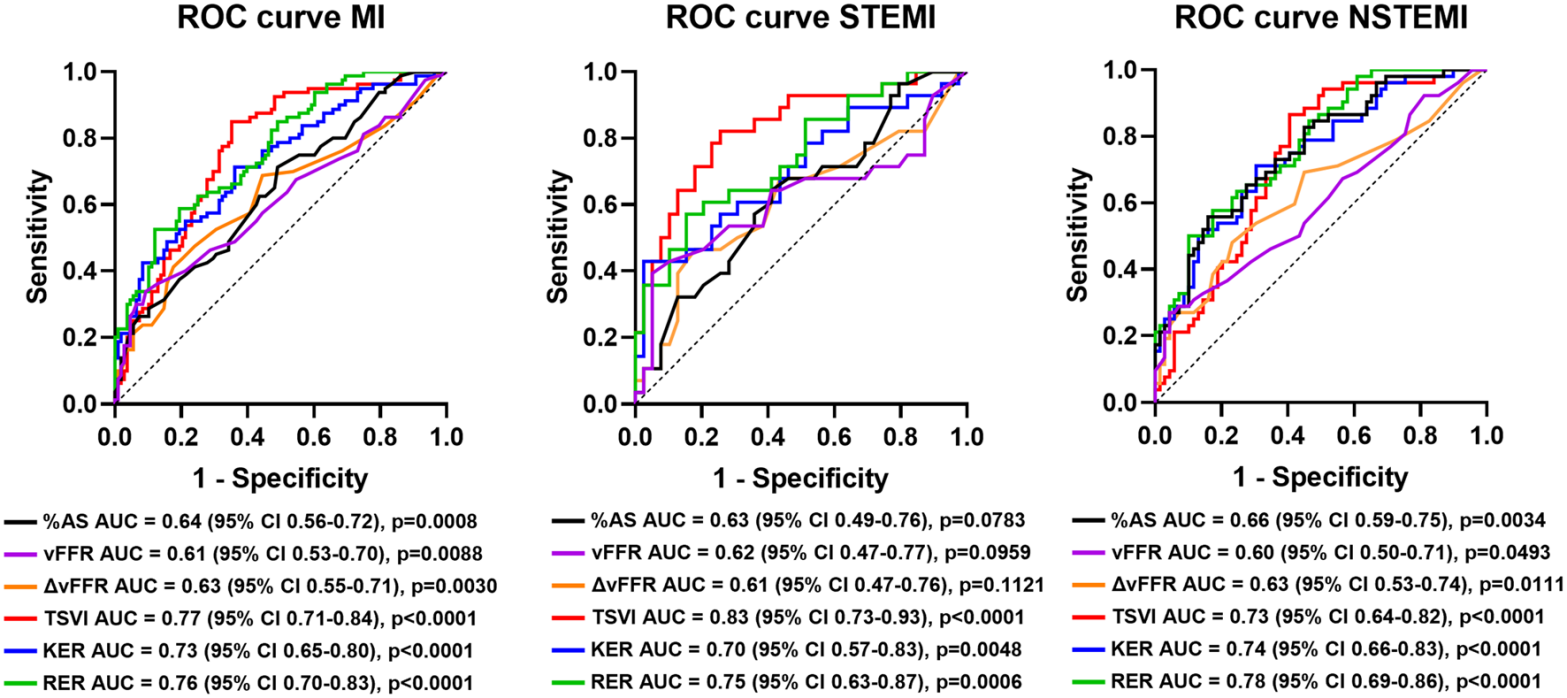
Receiver operating characteristic (ROC) curves for myocardial infarction (MI), ST-elevation MI (STEMI), non-ST-elevation MI (NSTEMI) for %AS, vFFR, ΔvFFR, TSVI, KER, and RER. The values of area under the curve (AUC) with 95% confidence interval (CI) and *p* values are reported for each quantity

Blood flow energy transformations had significant predictive capacity for future MI (KER: AUC = 0.73, 95% CI 0.65–0.80, *p* < 0.0001; RER: AUC = 0.76, 95% CI 0.70–0.83, *p* < 0.0001, Fig. 4).

The anatomical severity of the lesion, clinically quantified in terms of %AS, emerged as predictor for MI (AUC = 0.64, 95% CI 0.56–0.72, *p* = 0.0008), although (i) weaker (even if not significantly) than KER (DeLong test: *p* = 0.0620), and (ii) significantly weaker than RER (DeLong test: *p* = 0.0041).

The predictive capacity for MI of the functional severity of the lesion, clinically quantified in terms of vFFR (AUC = 0.61, 95% CI 0.53–0.70, *p* = 0.0088), was significantly weaker than RER and KER (DeLong test: *p* = 0.0045 and *p* = 0.0391, respectively). Focusing on the translesional pressure drop, ΔvFFR predicted MI with a moderate capacity (AUC = 0.63, 95% CI 0.55–0.71, *p* = 0.0030, Fig. 4), significantly weaker than RER (DeLong test: *p* = 0.0059) and not significantly different from KER (DeLong test: *p* = 0.0519). The WSS-based quantity TSVI emerged as strong predictor of future MI in the same population in a previous study (AUC = 0.77, 95% CI 0.71–0.84, *p* < 0.0001, Fig. 4) [6]. The predictive power for future MI of KER and RER was not significantly different with respect to TSVI (DeLong test: *p* = 0.2982 and *p* = 0.8927, respectively).

Analyzing separately lesions of patients presenting with future ST-elevation MI (STEMI), it emerged that (Fig. 4) (i) %AS (AUC = 0.63, 95% CI 0.49–0.76, *p* = 0.0783), vFFR (AUC = 0.62, 95% CI 0.47–0.77, *p* = 0.0959), and ΔvFFR (AUC = 0.61, 95% CI 0.47–0.76, *p* = 0.1121) were not significant predictors of future STEMI; (ii) high KER and high RER were significant predictors of future STEMI (AUC = 0.70, 95% CI 0.57–0.83, *p* = 0.0048; AUC = 0.75, 95% CI 0.63–0.87, *p* = 0.0006, respectively), with predictive power not significantly different (DeLong test: *p* = 0.0733 and *p* = 0.1664, respectively) from high TSVI (AUC = 0.83, 95% CI 0.73–0.93, *p* < 0.0001).

As for the lesions of patients presenting with future non-ST-elevation MI (NSTEMI), the findings of the analysis are summarized in Fig. 4: (i) high %AS (AUC = 0.66, 95% CI 0.59–0.75, *p* = 0.0034), low vFFR (AUC = 0.60, 95% CI 0.50–0.71, *p* = 0.0493), high ΔvFFR (AUC = 0.63, 95% CI 0.53–0.74, *p* = 0.0111) resulted in significant predictors for NSTEMI; (ii) high RER (AUC = 0.78, 95% CI 0.69–0.86, *p* < 0.0001) resulted in significant predictor for NSTEMI, stronger than %AS, vFFR, and ΔvFFR (DeLong test: *p* = 0.0194, *p* = 0.0092, and *p* = 0.0239, respectively); (iii) high KER (AUC = 0.74, 95% CI 0.66–0.83, *p* < 0.0001) resulted in a significant predictor for NSTEMI significantly stronger than vFFR (DeLong test: *p* = 0.0380) but not %AS and ΔvFFR (DeLong test: *p* = 0.0909 and *p* = 0.0759, respectively); (iv) KER and RER resulted in predictors for future NSTEMI not significantly different (DeLong test: *p* = 0.7535, *p* = 0.2886) from high TSVI (AUC = 0.73, 95% CI 0.64–0.82, *p* < 0.0001).

**Fig. 5:**
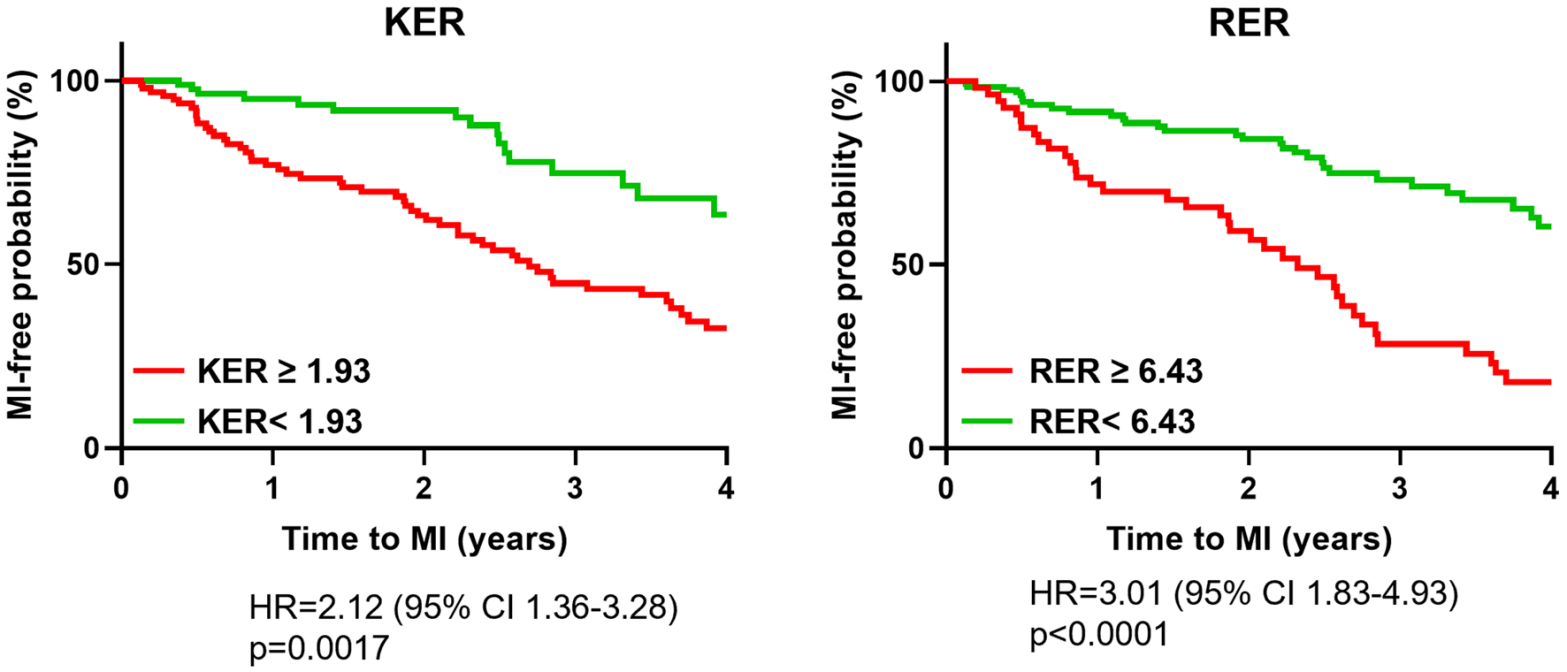
Time-to-event curves. Significantly divergent Kaplan–Meier curves for future myocardial infarction (MI) are represented at 4-year follow-up for KER and RER. Red and green curves refer to values above or below the threshold values obtained from the ROC analysis, respectively. Hazard ratio (HR) refers to the whole follow-up time interval (i.e., 5 years)

**Fig. 6:**
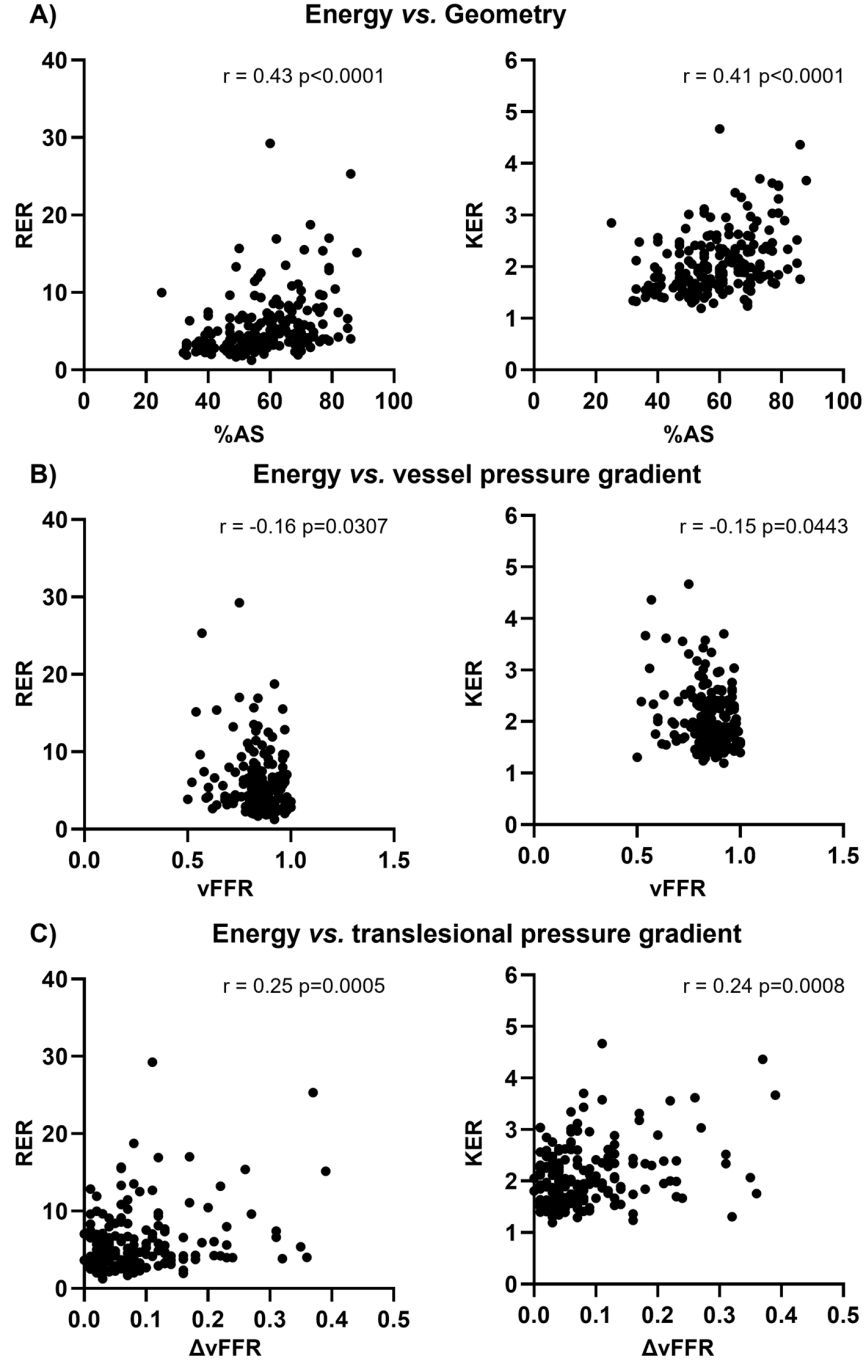
Scatter plots of rotational energy ratio (RER) and kinetic energy ratio (KER) *vs.* %AS (panel A), vFFR (panel B), and ΔvFFR (panel C). Spearman correlation coefficients (r) and corresponding *p*-values are reported

The time-to-event analysis demonstrated that KER and RER retain their predictive ability over time (Fig. 5). Baseline KER and RER value above the empirical threshold of 1.93 and 6.43 exhibited a twofold and threefold increase in the odds for future MI, respectively (hazard ratio [HR] = 2.12, 95% CI 1.36–3.28, *p* < 0.0017 for KER; HR = 3.01, 95% CI 1.83–4.93, *p* < 0.0001 for RER; Fig. 6).

### Lesion Geometry, Blood Flow Energy, and Wall Shear Stress

The nature of the relationships among the considered anatomical, functional, and hemodynamic quantities was explored. Significant positive correlations with %AS was observed for KER and RER (r = 0.41, *p* < 0.0001 and r = 0.43, *p* < 0.0001, respectively, Fig. 6A), indicating that kinetic and rotational energy increase as the lesion narrowing increases.

**Fig. 7:**
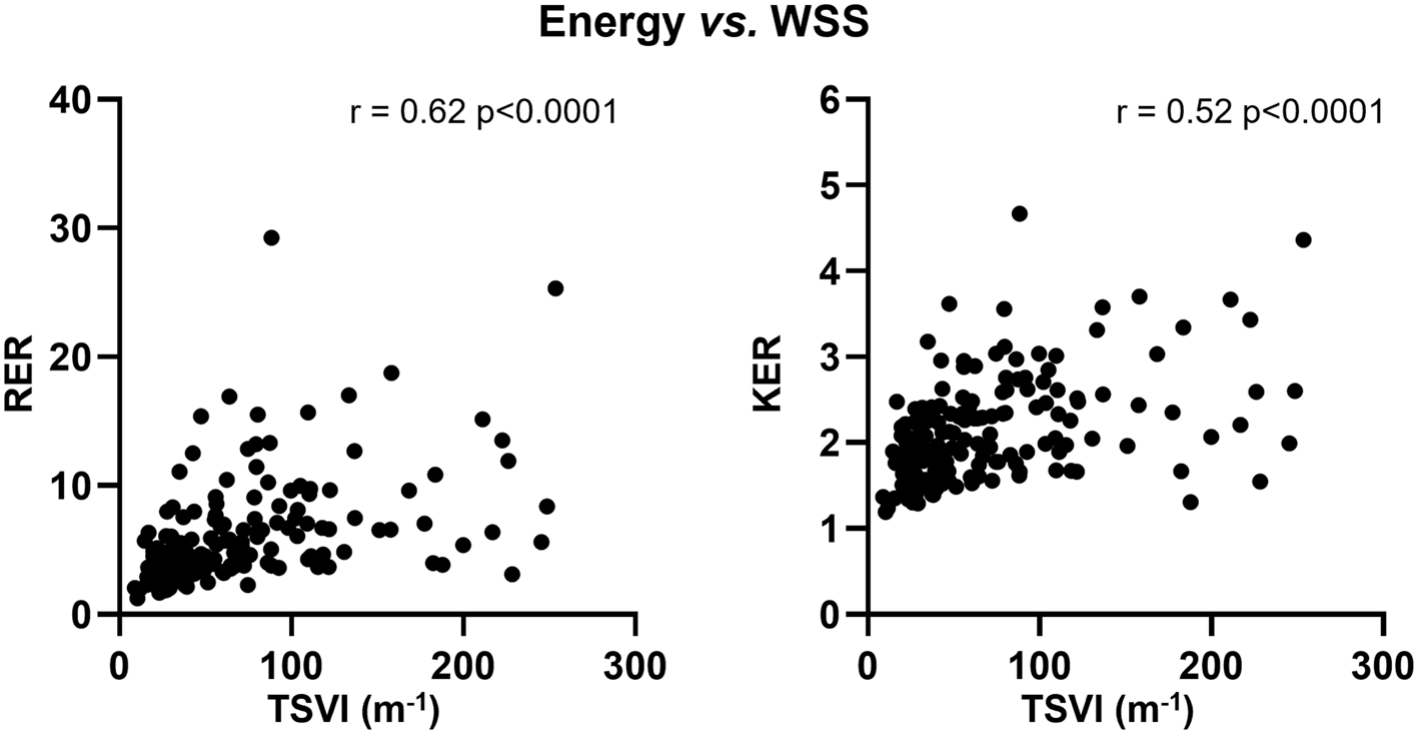
Scatter plots of rotational energy ratio (RER) and kinetic energy ratio (KER) *vs.* topological shear variation index (TSVI). Spearman correlation coefficients (r) and corresponding *p*-values are reported

A weak albeit significant inverse association of KER and RER with vFFR (r = − 0.16, *p* = 0.0307 and r = − 0.15, *p* = 0.0443, respectively, Fig. 6B) emerged. As for translesional pressures, KER and RER resulted moderately associated with ΔvFFR (r = 0.24, *p* = 0.0008 and r = 0.25, *p* = 0.0005, respectively, Fig. 6C). These results confirm the expected relationship between pressure gradients and kinetic energy and highlight the existence of a link also with rotational energy. A significant correlation was also found between blood flow energy-related quantities and TSVI (r = 0.52, *p* < 0.0001 and r = 0.62, *p* < 0.0001 for KER and RER, respectively, Fig. 7), suggesting that the higher the (kinetic, rotational) energy production within the converging flow segment of a lesion, the stronger the amount of near-wall flow disturbances (quantified in terms of TSVI).

## Discussion

The present study investigated intracoronary blood flow energy and related it to the risk of MI in the long term. The main findings of this study can be summarized as follows: (i) FC lesions presented more marked kinetic and rotational energy transformation (quantified by KER and RER, respectively) than NFC in the converging flow segment of the lesion; (ii) blood flow kinetic and rotational energy transformations strongly predicted MI at 5 years; and (iii) kinetic and rotational energies imparted to blood flow in the converging flow segment of the lesion emerged as predictors of future MI stronger than currently adopted angiography-based clinical quantities.

### Predictors of Myocardial Infarction

In the present study, it emerged that the net amount of blood flow energy transformations occurring in the proximal, converging flow part of the lesion reflected not only an increase of blood flow kinetic energy (KER > 1 at the cost of translesional pressure gradient generation ΔvFFR > 0, consistent with Bernoulli’s theorem that governs variations in velocity and pressure of fluids across tubular cross-sections), but also of its rotational component (RER > 1).

**Fig. 8:**
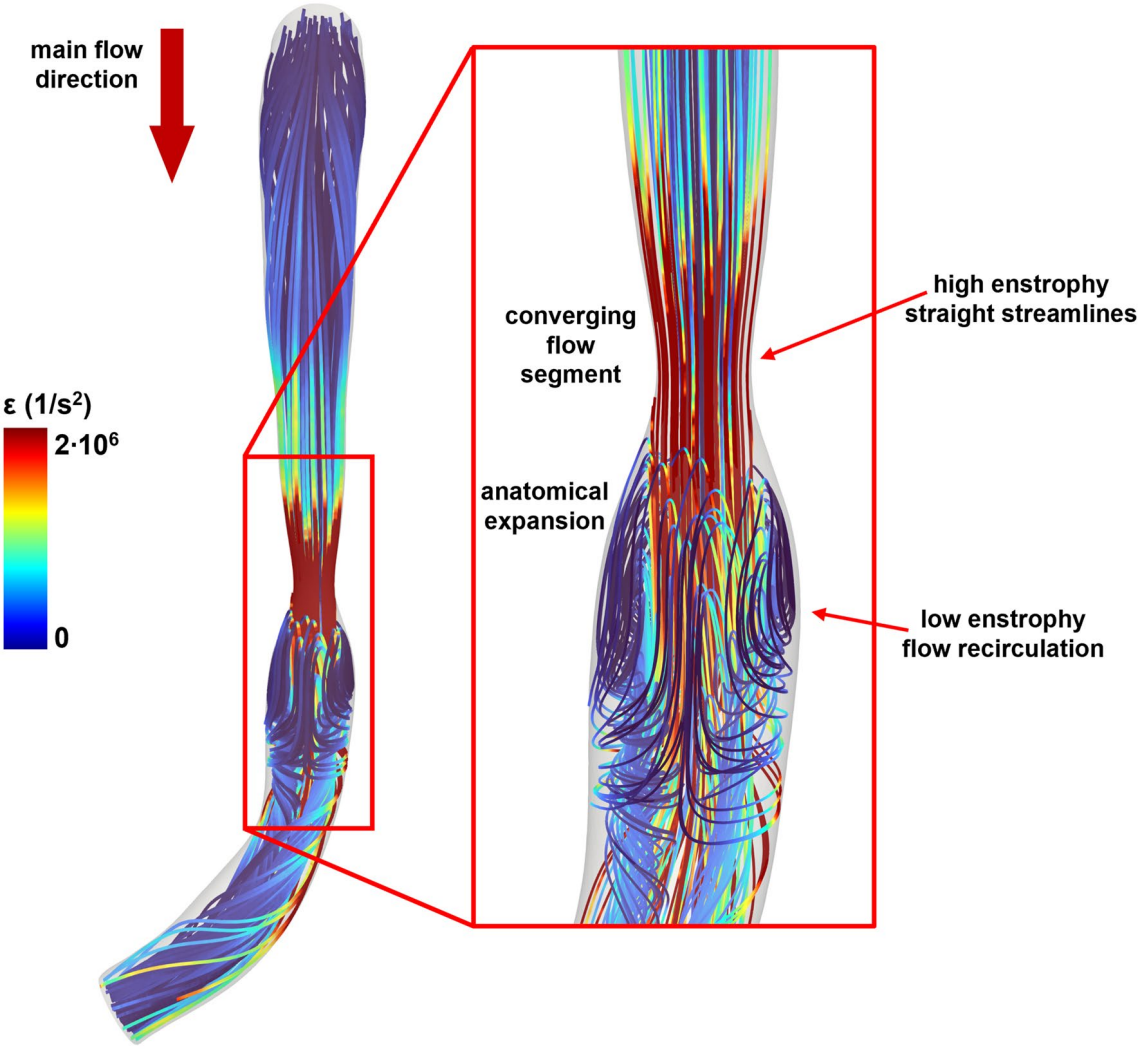
Velocity streamlines visualization color-colored with cycle-averaged local enstrophy (ε) values. Enstrophy is higher in the converging flow segment of the lesion, where the streamlines are basically straight, while lower values of enstrophy are found in the anatomical expansion of the vessel located distal to the MLA section, where flow recirculation regions are located

In the investigated patient study cohort, blood flow energy transformations were linked to coronary artery disease progression up to a clinically manifest event. Our findings support the evidence from previous studies on the pathophysiological significance of intracoronary helical blood flow, which results by the combination of translational and rotational blood flow motions [22, 23]. Moreover, the here reported findings expand and make sense of recent observations linking vorticity with mild intracoronary pressure gradients [8, 37] and with high shearing forces [37], providing a direct link with clinical outcomes that previous studies have only inferred. Beyond its rigorous mathematical definition, vorticity, intuitively recalling rotational motion [38], is the quantity largely adopted to detect vortices in the flow. This interpretation only partially distills the mathematical definition into the physics of fluids motion because in the coronary segments under study (i) enstrophy (and vorticity as well) increased as blood flows from the proximal to the MLA lesion section, and (ii) high enstrophy values were located toward the outer radial direction, i.e., close to the wall, with lower values in the region closer to the vessel axis (Fig. 3A) even if instantaneous streamlines are basically straight lines (Fig. 8). These observations clearly suggest that the observed enstrophy production was mainly the consequence of increased shearing in blood flow, caused by the progressive narrowing of the vessel lumen, and not by the onset of a purely rotational fluid motion. FC lesions presented a remarkably higher enstrophy production than NFC, which quantified in terms RER was predictor for future MI significantly stronger than %AS, vFFR, and ΔvFFR. Notably, the increase in blood flow kinetic energy, quantified in terms of KER, did not emerge as predictor for MI stronger than %AS and ΔvFFR.

In addition to the emerged strong predictive capacity of hemodynamic-related quantities for future MI, KER, RER, and TSVI were found to be significant predictors of both STEMI and NSTEMI. Being plaque erosion the predominant mechanism for NSTEMI [10], and being STEMI more likely associated with plaque rupture [26], in addition to the emerged strong predictive capacity of hemodynamic-related quantities for future MI, our findings suggest that a complex interplay of differently modulated biomechanical mechanisms and plaque phenotypes concur to adverse cardiac events.

### Interplay Between Blood Flow Energy and Wall Shear Stress

The hemodynamic features highlighted by KER and RER quantities were strong predictors for MI, with predictive power similar to that of the WSS-based quantity TSVI calculated over the whole lesion [6]. TSVI evaluated at lesion level [6] was associated with the blood flow energy produced in the proximal segment of the lesion (KER: r = 0.52, *p* < 0.0001 and RER: r = 0.62, *p* < 0.0001). Since the distal segment of the lesion was characterized by the highest TSVI values [6], our findings suggest that marked kinetic and rotational energy transformations occurring in the proximal segment of the lesion exacerbate flow disturbances not only at the level of the lesion throat, but also in the anatomical expansion of the vessel located distal to the MLA section. This may reflect into a marked variability in the contraction/expansion action exerted by WSS on the endothelium along the cardiac cycle, which in turn may impact on intracellular and cell-cell tensions promoting shrinking/widening of cellular gaps [6, 19–21]. The observed distribution of high enstrophy values in the near-wall region, its emerged dependence on shearing and the significant direct relationship with TSVI also suggest a direct link between vorticity production and WSS-related biological adverse events, which is even more evident in lesions with larger area reduction.

These findings have direct clinical implications. In fact, bulk flow-based quantities like KER and RER may facilitate the implementation and acceptance of 3D QCA-based CFD analyses for the risk assessment of MI in real-world clinical settings with respect to WSS-based quantities. This is because a methodology based on clinical QCA might oversimplify the heterogeneous subtleties of lumen topography which have the potential to affect WSS [31]. The proposed bulk flow quantities are inherently less sensitive than WSS-based quantities to the accuracy of the reconstruction of the complex lumen topography. Moreover, they hold promise for future implementation in a clinical setting also utilizing widely adopted clinical imaging techniques such as 4D flow magnetic resonance imaging [3].

### Limitations of the Study

There are some limitations that could weaken the findings of this study. The first limitation is related to the retrospective nature of the study, which does not allow controlling potential confounding factors, e.g., the administration of intracoronary nitroglycerin before angiographic images acquisition. A second limitation might be the adopted clinical imaging technology (i.e., coronary angiography), from which no direct information about plaque composition can be derived [11]. Lastly, the implementation of computational models unavoidably requires the introduction of assumptions/idealizations of the problem under study. Examples regarding the assumption of rigid vascular walls, the absence of cardiac-induced motion and coronary displacement, or the lack of personalized flow measurements are discussed in previous studies [5, 12, 16, 35]. All these assumptions/idealizations contribute to the budget of uncertainty affecting the outcome of a simulation. However, such an uncertainty is counterbalanced by the several advantages offered by in silico modeling. Among them and in relation to the context of use in the present study, we recall here that in silico modeling is cost effective in providing quantities that currently cannot be measured in vivo in a repeatable and user-independent manner with other technologies. Hence, computer models inevitably produce results affected by uncertainty, similarly to the other technologies currently adopted. However, this does not detract from the usefulness of computational hemodynamics in providing mechanistic insights and predictions on disease progression that can inform decision making and guide further research. *Stricto sensu*, uncertainty does not affect only personalized in silico models of coronary flows, but also the technologies currently applied in the clinical practice by cardiologists. In perspective, the future adoption of the in silico “technology” for coronary hemodynamics quantitative analysis is purely a problem of standardization of the methodology to be implemented [29]. Standardization should rest on the identification of minimal requirements that should be universally adopted, in analogy with (and hopefully within) clinical guidelines.

Of relevance, KER and RER present the important advantage of being normalized quantities, by construction. Thus, they are expected to be only modestly sensitive to the flow rate values imposed as boundary condition [16] but driven mainly by the lesion narrowing.

### Conclusions

The findings of this study proved that the blood flow kinetic and rotational energy transformations induced by the lumen narrowing in correspondence of coronary atherosclerotic lesions are significant predictors for future MI. The amount of blood flow energy transformations outperformed the angiography-based anatomical and functional quantities currently adopted in clinical cardiology in predicting future MI, in particular energy transformations related to rotational energy. Moreover, blood flow energy transformations exhibited a predictive performance comparable to WSS-based quantities. These findings warrant and encourage further studies of blood flow energy transformations in patients with coronary artery disease to support KER and RER clinical translation in screening lesions culprit of future MI.

As computer simulations are currently making inroads in the interventional cardiology workspace, e.g., to provide FFR [32] non-invasively or WSS [6], the quantitative analysis of intravascular flow features such as the one presented here would enrich the arsenal of tools available for clinical decision making.

### Supplementary Information

Below is the link to the electronic supplementary material.

Supplementary file1 (PDF 124 kb)
