# Supplementary material for: Blood Flow Energy Identifies Coronary Lesions Culprit of Future Myocardial Infarction
Source: Ann Biomed Eng. 2023 Sep 21;52(2):226–38. doi: 10.1007/s10439-023-03362-3 (PMC11252236; doi:10.1007/s10439-023-03362-3)
Supplement: Supplementary file 1 — Supplementary file1 (PDF 124 kb) [file 10439_2023_3362_MOESM1_ESM.pdf]

Blood flow energy identifies coronary lesions culprit of future myocardial infarction

-Supplementary Materials-

Maurizio Lodi Rizzini[1], Alessandro Candreva[1,2], Valentina Mazzi[1], Mattia Pagnoni[3], Claudio Chiastra[1], Jean-Paul Aben[4], Stephane Fournier[3,5], Stephane Cook[6], Olivier Muller[3], Bernard De Bruyne[7], Takuya Mizukami[7], Carlos Collet[7], Diego Gallo[1], Umberto Morbiducci[1]

1 Polito[BIO]Med Lab, Department of Mechanical and Aerospace Engineering, Politecnico di Torino, Torino, Italy

2 Department of Cardiology, Zurich University Hospital, Zurich, Switzerland

3 Department of Cardiology, Lausanne University Hospital, Lausanne, Switzerland

4 Pie Medical Imaging BV, Maastricht, the Netherlands

5 Dept. of Advanced Biomedical Sciences, University of Naples Federico II, Naples, Italy

6 Department of Cardiology, HFR Fribourg, Fribourg, Switzerland

7 Cardiovascular Center Aalst, OLV-Clinic, Aalst, Belgium

Address for correspondence:

Prof. Umberto Morbiducci

PoliTo[BIO]Med Lab, Department of Mechanical and Aerospace Engineering

Politecnico di Torino

Corso Duca degli Abruzzi 24

10129 Turin, Italy

Tel.: +39 011 0906882

E-mail: umberto.morbiducci@polito.it

Supplementary Methods

Three-dimensional (3D) reconstructions of coronary arteries were performed using the CAAS Workstation WSS software (Pie Medical Imaging, Maastricht, the Netherlands) according to the following steps:

1) importation of two X-ray coronary angiography projections as DICOM (Digital Imaging and Communications in Medicine) files with at least 30° difference in rotation/angulation;

2) selection of the end-diastolic frame from cineangiographies. If ECG triggering is present, the selection is performed automatically, while if it is not present a manual frame selection is performed;

3) segmentation of the vessel, which is started on one angiographic projection by a semi-automatic process that employs either a manually drawn initial or an automatic pathline. The two mentioned processes are both initiated by means of two user-defined points identifying the coronary segment of interest: one proximal (P) and one distal (D). The automatic pathline is calculated by a wave propagation algorithm[2]. Subsequently, automatic contour detection is performed via a validated algorithm featuring improved analysis for small vessel lumens[1]. In short, the contour detection applies a minimal cost algorithm based on the differences in the local grey scale and dynamically integrates video-densitometric information from the image into the segmentation;

4) the region of interest is automatically indicated in the other selected angiographic projection, thereby assisting the user in correctly placing the delimiter points around the coronary segment of intertest, which is then contoured with the same algorithm as described above;

5) to obtain an accurate and robust 3D reconstruction of the coronary artery, a correction for system distortion introduced by image isocenter offset is required. This correction is performed by identifying a common image point (CIP) in both angiographic projections, which represents a corresponding anatomical landmark between the selected angiographic projections. Within CAAS Workstation WSS software, this correction is automatically performed based on a correlation algorithm between the video-densitometric intensities obtained from the detected coronary artery in both angiographic projections;

6) the 3D centerline of the coronary artery is reconstructed by means of an adaptive 3D epipolar geometry-based algorithm[3];

7) the 3D luminal cross-sections of the vessel are reconstructed assuming an elliptical model, by using the luminal diameters of the corresponding 2D cross sections and their spatial orientations to define the ellipse axes;

8) finally, the vessel centerline and the elliptical cross-sections are used to build up a 3-D triangular surface mesh.

Supplementary References

1. Girasis, C., J. C. H. Schuurbiers, Y. Onuma, J. P. Aben, B. Weijers, M. A. Morel, J. J. Wentzel, and P. W. Serruys. Advances in two-dimensional quantitative coronary angiographic assessment of bifurcation lesions: Improved small lumen diameter detection and automatic reference vessel diameter derivation. *EuroIntervention* 7:1326–1335, 2012.

2. Janssen, J. P., G. Koning, P. J. H. De Koning, J. C. Tuinenburg, and J. H. C. Reiber. A novel approach for the detection of pathlines in X-ray angiograms: The wavefront propagation algorithm. *Int. J. Cardiovasc. Imaging* 18:317–324, 2002.

3. Onuma, Y., C. Girasis, J. P. Aben, G. Sarno, N. Piazza, C. Lokkerbol, M. A. Morel, and P. W. Serruys. A novel dedicated 3-dimensional quantitative coronary analysis methodology for bifurcation lesions. *EuroIntervention* 7:629–635, 2011.

Supplementary Figure

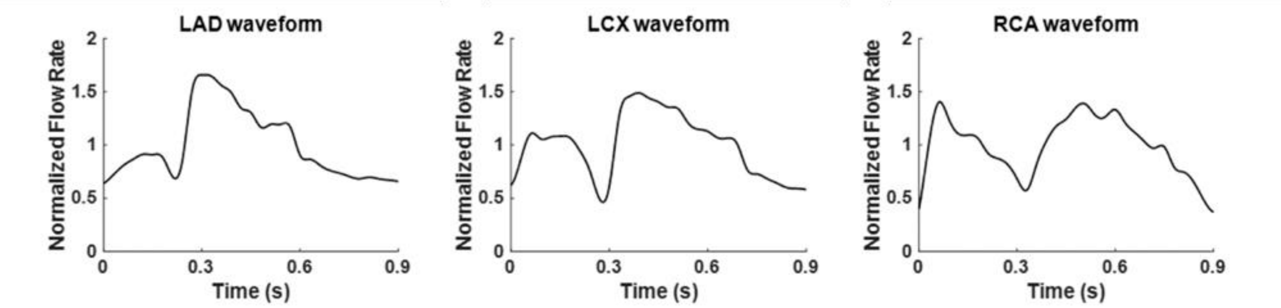

Figure S1. Normalized flow rate waveforms for (from left to right): left anterior descending (LAD), left circumflex (LCX), and right coronary artery (RCA).

Supplementary Table

Supplementary Table 1. Clinical characteristics (N= 80)	
Age, years	70.3 ±12.7
Female	23 (28.7%)
Type of MI	
- NSTEMI	52 (65.0%)
- STEMI	28 (35.0%)
Treatment	
- PCI	78 (97.5)
- CABG	1 (1.3%)
- Medical	1 (1.3%)
Time from baseline ICA, months	25.9 ± 17.7
- 1 ICA before MI, n	67 (83.7%)
- ≥ 2 ICA before MI, n	13 (16.3%)
Hypertension	61 (76.3%)
Hyperlipidemia	63 (78.8%)
Diabetes mellitus	20 (25.0%)
Insulin therapy	7 (8.8%)
Smoking	21(26.3%)
LVEF < 55%	18 (22.5%)
Reduced kidney function*	18 (22.5%)
Prior PCI	37 (46.3%)
Prior stroke	13 (16.3%)
Prior PVD	18 (22.5%)
Aspirin	61 (76.3%)
Statin	72 (90.0%)
P2Y12 inhibitors	15 (18.8%)

Clinical characteristics of the studied population at the time of the acute myocardial infarction. Coronary artery bypass graft, CABG; Invasive coronary angiography, ICA; Left ventricle ejection fraction, LVEF; Myocardial infarction, MI; non-ST segment elevation myocardial infarction, NSTEMI; Percutaneous coronary intervention, PCI; Peripheral vascular disease, PVD; ST segment elevation myocardial infarction, STEMI.
* estimated glomerular filtration rate (eGFR) < 60 ml/min/1.73 m^2.
